# Study on decarbonization trajectories and policy implications for China: A comparative analysis of carbon peak nations

**DOI:** 10.1371/journal.pone.0308394

**Published:** 2024-08-06

**Authors:** Su Xu, Jun Wang

**Affiliations:** Department of Landscape Planning and Design, School of Art Design and Media, East China University of Science and Technology, Shanghai, China; Shandong University of Technology, CHINA

## Abstract

Amidst escalating global concerns over climate change and the pressing need for sustainable development, this study conducts a comparative analysis across 24 nations that have successfully achieved carbon peaking, evaluating their socioeconomic characteristics and carbon reduction strategies. Simultaneously, it examines China’s policy evolution and strategic responses within the context of its economic and urban development. The analysis reveals that countries with successful carbon peak outcomes typically exhibit high GDP per capita and advanced urbanization rates. Critical to their success are comprehensive adjustments in energy consumption structures and industrial transformation, which are supported by robust environmental policies and technological innovation. The study categorizes global carbon reduction policies into three primary categories and seven sub-categories, reflecting the dynamic evolution of policy approaches driven by global climate agendas and varying stages of national development. Strategies including legal frameworks, carbon pricing mechanisms, international cooperation, and technological innovation are critically assessed for their potential to refine China’s carbon policies. Significant challenges in policy implementation are identified, particularly in aligning ambitious environmental strategies with economic objectives and managing transition costs in critical sectors such as energy and transportation. The study emphasizes the necessity of a phased policy implementation approach, which begins with enhancing public and corporate environmental awareness, advances through the promotion of low-carbon technologies, and concludes with the establishment of stringent legal and regulatory frameworks.

## 1.Introduction

China released its policy commitment on carbon peaking in September 2020, pledging to achieve carbon emissions peaking around 2030 and striving for carbon neutrality around 2060. This policy demonstrates the Chinese government’s recognition of climate change as a worldwide issue and showcases China’s perspective and dedication to tackling it. China has the potential to achieve carbon peaking through a more secure and efficient energy system, improved economic development, and a continuously optimized carbon emission structure. However, in contrast to developed countries and those that have already reached their carbon peaks, China’s process of promoting carbon emission reduction started relatively late and faces multiple pressures such as management of overall peak emissions and the restructuring of the economic framework. China faces more significant challenges in reducing carbon emissions compared to wealthy countries, as it is under greater pressure to achieve carbon neutrality within a shorter timeframe and with more demanding goals [1, 2] Carbon peaking is a matter of worry not just for the energy and industrial sectors, but also for urban and rural construction, transportation, and other related areas. Examining the socio-economic attributes and policy trajectories of countries that have already reached their peak can offer significant benchmarks for China and other nations in search of appropriate models and strategies to attain carbon peaking.

Prior research predominantly adopted qualitative analysis and case studies, approaching from multiple professional perspectives to synthesize the carbon reduction experiences of countries that have successfully reached carbon peaking. Wang Kun et al. (2023) examined the policy experiences of the United States and Germany during their carbon peaking processes, focusing on the structure of energy consumption [3]. Ma Haiyun et al. (2023) focused on the perspective of technological innovation and employed the "Technology-Organization-Environment" framework to discern typical trends in the adoption and application process of low-carbon technological innovation in countries that have taken the lead in reaching carbon peaks [4]. Zhang Jianping et al. (2023) analyzed the cooperation practices of major developed countries and economies, including the European Union, Japan, and the United States, to gain insights into international collaboration in tackling climate change [5]. Mao Xinhui et al. (2024) documented the spatial planning strategies implemented by industrialized countries and organizations as a response to climate change [6]. In addition, certain researchers have embraced a contemplative viewpoint, examining negative examples where excessively forceful carbon reduction programs in specific Western nations and areas resulted in economic, social, and financial hazards. Consequently, they have scrutinized these instances of failure and the accompanying risks [3].

Previous studies have recognized that, based on the Kuznets curve, there is an anticipated alignment between environmental quality and economic development at later stages of economic growth. This alignment is characterized by a stronger influence of low-carbon technologies and policy innovations on reducing carbon emissions in societies with higher levels of economic development. However, existing researches are mostly based on the studies of individual or a small number of cases. There is a lack of comprehensive assessments that provide detailed characteristics of nations that have successfully reached carbon peaking. Zhang Nan et al. (2021) performed a trend analysis on CO_2_ emissions and socio-economic data from 219 nations and regions. They focused on summarizing the features of per capita GDP, the proportion of the tertiary sector, and fossil energy architectures in countries that have already reached their emission peaks [7]. Zhang Mengnan and his colleagues (2024) examined the phase features of prominent nations and regions, such as the European Union, the United States, and Japan, when they reached the point of reaching carbon peaking [1].

When examining the various elements that affect the achievement of carbon emission targets, research emphasizes the significant influence of aspects such as economic activities, social policies, technical innovations, and regulatory contexts on carbon emissions. Firstly, there are intricate connections between economic issues such as remittances and economic growth and the levels of CO_2_ emissions. Research conducted by Shahbaz et al. (2021) [8] and Bashir et al. (2020) [9] demonstrates that remittances and agricultural value addition typically have a positive impact on reducing emissions. Conversely, financial development and industrial growth tend to result in higher emissions. In addition, the manner in which energy is consumed has a substantial impact on the environment. Sohail et al. (2023) [10] highlight that adopting clean energy sources and lowering reliance on old energy sources are effective approaches to improve environmental sustainability. Regarding social matters, the clear good effects of education and gender equality on environmental conservation are evident. The study conducted by Wang et al. (2021) [11] demonstrates that enhancing women’s education levels and expanding employment opportunities contribute to a decrease in CO_2_ emissions. Ullah et al. (2020) [12] highlight the significance of tackling energy poverty and improving education and employment levels as essential approaches to foster economic development and environmental sustainability. Moreover, the achievement of renewable energy projects depends on efficient organizational communication and technological advancement. Khan et al. (2022) [13] and Ali et al. (2022) [14] emphasize the crucial roles of supportive leadership and technological factors in promoting project success. Additionally, Ahmed et al. (2020) [15] highlight that strong organizational support can alleviate the adverse effects of toxic work environments and high job stress on energy projects. Improving energy efficiency and environmental sustainability are seen as essential goals that can be achieved through digital transformation and regional economic cooperation, both at the technological and regulatory levels. In their study, Shahbaz et al. (2024) [16] investigated the utilization of digitalization in the mining industry, showcasing the capacity of technological advancements to mitigate the squandering of resources. According to a study conducted by Zhang et al. (2018) [17], economic collaboration, such as the Silk Road Economic Belt, can help member nations integrate their economies and environment, leading to sustainable growth. These studies highlight the importance of taking into account the interaction of economic, social, technological, and policy aspects while aiming to reach maximum carbon emission reduction goals. By incorporating these ideas, a more efficient and successful approach to addressing global climate change concerns can be developed.

These studies indicate that the effectiveness of low-carbon experiences depends on appropriate environmental and contextual factors, otherwise, the logic of its effectiveness cannot be replicated in China. However, the socio-economic data analysis in nations that have reached their peak in carbon emissions is lacking in current study. This study attempts to refine the features of carbon peak countries and analyze their general characteristics from several angles, including socio-economic and energy ecology.

This study aims to thoroughly investigate the occurrence of carbon peaking, taking into account socio-economic factors and government actions. thereby extending the application and depth of the Environmental Kuznets Curve (EKC) theory. The EKC hypothesis traditionally explains the dynamic correlation between economic development and environmental degradation. It suggests that environmental pollution tends to rise during the initial phases of economic expansion and then declines as the economy further expands. This research employs a theoretical framework to systematically compare and analyze various aspects of data from 24 countries that achieved carbon peaking after 1991. This analysis covers various factors, including population size, levels of economic and urban growth, carbon emission levels, trends in industrial structure, and energy consumption, as well as environmental indicators.

The study thoroughly analyzes the performance of these countries based on indicators such as GDP per capita, urbanization rates, energy consumption structures, and industrial transformation. These indicators directly demonstrate the relationship between economic growth and environmental effect, as proposed by the EKC theory. This research employs quantitative studies to uncover the phased characteristics of low-carbon development. It also examines how different countries have successfully achieved carbon peaking through policy and technical advances at different stages of their economic and environmental progress. In addition, the paper briefly examines how international collaboration and technical advancements can expedite the achievement of the expected turning points for environmental degradation as suggested by the Environmental Kuznets Curve (EKC).

By prioritizing these analyses, the research not only confirms the widespread usefulness of the EKC theory in global efforts to reduce carbon emissions, but also establishes a strong theoretical and empirical basis for developing specific carbon reduction strategies that take into account the economic and social characteristics of different countries. These measures help to better tackle the issues of global climate change and provide useful experience and policy ideas for China and other countries in reaching carbon peaking.

## 2.Materials and methods

### (1) Data collection

The primary dataset for this analysis was sourced from the World Resources Institute (WRI), which provided comprehensive information on carbon emissions, urbanization rates, GDP per capita, and energy consumption for 24 countries that have achieved carbon peaking since 1991. Additional economic and environmental data were obtained from the World Bank and the International Energy Agency to ensure the robustness of socioeconomic indicators and energy profiles.

### (2) Analytical framework

A multidimensional comparative analysis was conducted to assess the factors influencing carbon peaking. This framework involved:

Descriptive Statistics: Key socioeconomic and environmental indicators were summarized using measures of central tendency (mean, median) and dispersion (standard deviation, range) to characterize the profile of carbon-peaked countries.

Regression Analysis: To examine the relationship between carbon peaking and various independent variables, multiple linear regression models were employed. These models helped to identify significant predictors of carbon emissions peaks.

Cluster Analysis: Countries were grouped based on similarities in socioeconomic status, energy consumption patterns, and carbon peaking timelines using hierarchical clustering techniques. This method facilitated the identification of common attributes among countries with similar carbon peaking profiles.

### (3) Selection criteria

Based on the availability of data, this study selected 24 countries that have achieved carbon peaking since 1991 for sample analysis. The countries included in this research vary from former Soviet and Eastern European nations that have been established for a long time to more recent Asian countries such as Japan and South Korea. These countries have significant differences in terms of economic development, levels of industrialization, and policies for reducing carbon emissions. Therefore, they offer a wide and relevant range of perspectives for this research. The inclusion of countries with varying economic scales and sizes, ranging from major industrialized nations such as the USA and Japan to smaller countries like Ireland and New Zealand, enhances our comprehension of the carbon peaking process within diverse socioeconomic and political situations. This diversity is essential for developing strategies that are in line with global norms in energy transformation and the adoption of low-carbon technology in China. The study’s timeline, spanning from 1991 to 2020, enables us to examine the progression of carbon peaking from its early phases to current trends. This is crucial for studying the enduring shifts and periodic variations in the carbon peaking process.

## 3. Results

### 3.1 Overview of countries that have reached their peak in carbon emissions

According to data from the World Resources Institute (WRI) in 2020, 53 countries worldwide have reached the point of carbon peaking, as seen in Table 1. These countries collectively account for almost 40% of the total global carbon emissions. Out of these, 19 countries, such as Germany and Russia, achieved their highest levels of carbon emissions throughout the 1990s. Between 1991 and 2000, fourteen countries, including the United Kingdom, France, Denmark, and Sweden, successfully reached their peak carbon emissions. Between 2001 and 2010, an additional 16 countries, including Canada, the United States, and Portugal, reached their highest levels of carbon emissions. Japan and South Korea, along with four other countries, achieved their peak carbon emissions between 2011 and 2020. China, Singapore, and Mexico have committed to reaching carbon peaking by 2030 [8]. By then, it is anticipated that the carbon emissions from all countries that have reached their highest point will make up roughly 60% of the global carbon emissions.

**Table 1:** Countries that have achieved or committed to achieving their carbon emissions peak by 2020.

Year	Countries that have achieved or pledged to peak carbon emissions
Pre-1990	Azerbaijan, Belarus, Bulgaria, Croatia, Czech Republic, Estonia, Georgia, Germany, Hungary, Kazakhstan, Latvia, Moldova, Norway, Romania, Russia, Serbia, Slovakia, Tajikistan, Ukraine
1991–2000	France, Lithuania, Luxembourg, Montenegro, United Kingdom, Poland, Sweden, Finland, Belgium, Denmark, Netherlands, Costa Rica, Monaco, Switzerland
2001–2010	Ireland, Micronesia, Austria, Brazil, Portugal, Australia, Canada, Greece, Italy, Spain, United States, San Marino, Slovenia, Liechtenstein, Iceland, Cyprus
2011–2020	Japan, South Korea, Malta, New Zealand

Among the countries that achieved carbon peaking in 1990 and earlier, some were from the former Soviet Union and Eastern Europe. One contributing element to their early achievement of this environmental milestone was the economic crisis. Simultaneously, several European countries diligently implemented carbon reduction programs and facilitated a shift towards a low-carbon economy, successfully reaching their peak carbon emissions [9]. From 1991 to 2010, there was a significant increase in national efforts to achieve carbon peak, with 30 countries reaching their highest point in carbon emissions. Except for Costa Rica, the majority of the 14 nations that reached their carbon peak between 1991 and 2000 were mainly European, with a notable presence from Western and Northern Europe. Between 2001 and 2010, a total of 16 countries reached their highest levels of carbon emissions, mainly located in North America, Southern Europe, Oceania, and South America. Brazil and the United States, which are significant contributors to carbon emissions, reached their peaks in 2004 and 2007 respectively. Out of the four countries that committed to reaching their peak carbon emissions by 2020, Japan and South Korea, two Asian countries successfully achieved this goal by 2013.

Table 2 shows that in the year of carbon peaking, the level of economic development and urbanization in the countries involved was relatively high., with an average urbanization rate of 75.63% and an average per capita GDP of $30,006.72. Carbon emission levels exhibited fluctuations, with an average emission rate of 0.35 kg per unit of GDP and an average emission rate of 9.69 tons per person. In terms of industrial structure, the tertiary industry is dominant in all countries with the average share of agricultural and industrial added value being 2.76% and 25.40% respectively. The energy consumption gap among these nations was significant, with an average energy intensity of $10.05 per GDP kilogram and a mean fraction of fossil fuel use of 75.61%. Significant differences were also noted in the biological environment of these countries, with an average amount of arable land of 16.29% and an average forest coverage of 36.14%.

**Table 2:** Descriptive statistical results of relevant indicators for 24 countries that have reached their carbon peaks.

Indicators	Urbanization Rate (%)	Per Capita GDP (USD)	Carbon Emission per Unit of GDP (kg/USD)	Per Capita CO2 Emissions (Tons)	Agriculture Value Added Proportion (%)	Industry Value Added Proportion (%)	Energy Consumption per Unit of GDP (USD/kg)	Fossil Fuel Energy Consumption (%)	Arable Land Proportion (%)	Forest Area Proportion (%)
Maximum Value	96.85	56943.37	0.64	19.06	11.11	34.45	18.32	98.53	54.73	72.57
Minimum Value	52.21	3637.31	0.16	1.30	1.05	17.90	3.24	13.17	1.25	0.42
Average Value	75.63	30006.72	0.35	9.69	2.76	25.40	10.05	75.61	16.29	36.14
Median Value	77.93	28935.39	0.32	9.31	2.20	25.59	9.98	83.65	14.81	31.90

Considering the substantial variations in economic scale and population size between countries, and recognizing that population structure plays a crucial role in the rise of carbon emissions [18], a comprehensive analysis of carbon peaking in different nations requires a more precise methodology. Hence, the subsequent section classifies the 24 countries that have attained carbon peaking into specific population size categories. This classification seeks to examine the common qualities and trends that are linked to carbon peaking.

### 3.2 Regression analysis and findings

This study meticulously developed a regression model to provide a robust analytical framework that captures the complex interplay between socio-economic development and environmental impact. Considering a range of determinants such as demographic dynamics, socio-economic structure, industrial development, and energy emissions, and evaluating the model’s goodness-of-fit alongside the statistical significance of the variables, we meticulously conducted multiple iterations of testing. This process led us to select four indicators—Urbanization Rate (UR), Annual Per Capita GDP (APCG), Proportion of Industrial Added Value (PIAV), and Fossil Fuel Energy Consumption (FFEC) to explain the variations in Per Capita CO_2_ Emissions (PCCE)(see Eq 1).


$$PCCE=\beta_{0}+\beta_{1}\times UR+\beta_{2}\times APCG+\beta_{3}\times PIAV+\beta_{4}\times FFEC+\epsilon$$ (1)

To ensure the model’s reliability and validity, comprehensive statistical diagnostics were performed, addressing multicollinearity, heteroscedasticity, and autocorrelation (see Table 3). To combat multicollinearity, a natural logarithm transformation was applied to each variable, enhancing the model’s stability and interpretability as illustrated in Eq (2):

$$\ln\;PCCE=\beta_{0}+\beta_{1}\times \ln(UR)+\beta_{2}\times \ln\left(APCG\right)+\beta_{3}\times \ln\left(PIAV\right)+\beta_{4}\times \ln(FFEC)+\epsilon$$ (2)

**Table 3:** Coefficients^a^.

Model		Unstandardized Coefficients		Standardized Coefficients	t	Sig.	Collinearity Statistics	
		B	Std. Error	Beta			Tolerance	VIF
1	(constant)	-13.046	2.226		-5.859	.000		
	Urbanization Rate (Proportion of Urban Population to Total Population)	.774	.367	.204	2.105	.049	.864	1.158
	Annual Per Capita GDP (USD)	.726	.088	.775	8.271	.000	.926	1.080
	Proportion of Industrial Added Value	.693	.355	.177	1.951	.066	.989	1.011
	Fossil Fuel Energy Consumption	.523	.134	.365	3.902	.001	.929	1.077

a. Dependent Variable: Per Capita CO_2_ Emissions

This transformation not only mitigates multicollinearity but also better reflects the multiplicative impact of these variables on CO_2_ emissions. The elasticity represented in the model allows for an understanding of how percentage changes in the predictors correspond to percentage changes in CO_2_ emissions, thereby enhancing the model’s predictive accuracy and relevance.

The model demonstrated a strong predictive power, as reflected by an R-squared value of 0.919, indicating that approximately 91.9% of the variability in per capita CO_2_ emissions is explained by the predictors included. The adjusted R-squared value of 0.845 further confirms that the model is highly effective, adjusting for the number of predictors.

The ANOVA results show a significant model fit (F = 25.982, p < .000), validating the overall statistical significance of the regression model. This supports the hypothesis that key socioeconomic and environmental factors significantly influence CO_2_ emissions.

Specifically, the regression coefficients suggest:

**Urbanization rate:** The model estimates a significant negative impact on CO_2_ emissions (B = -13.046, p < .000), implying that increases in urbanization rate are associated with reductions in per capita CO_2_ emissions. This result is particularly robust, with a notable t-statistic of -5.859.

**Annual per capita GDP:** This variable showed a positive correlation with CO_2_ emissions (B = .726, p < .000), indicating that higher GDP per capita is associated with increased emissions.

**Proportion of industrial added value:** Although this predictor approached significance (B = .693, p = .066), suggesting a potential positive effect on emissions, it did not reach conventional levels of statistical significance.

**Fossil fuel energy consumption:** Demonstrated a significant positive effect (B = .523, p = .001), confirming that higher fossil fuel consumption substantially increases CO_2_ emissions.

The Variance Inflation Factor (VIF) values for all predictors were well below the commonly used threshold of 10, suggesting no concerning levels of multicollinearity among the variables. The highest VIF observed was 1.158, indicating good tolerance and minimal inflation of variance in the regression estimates.

### 3.3 Analysis of characteristics of countries that have peaked in carbon emissions

The 24 countries that have reached carbon peaking were categorized into four groups based on their population size at the time of peaking, as shown in Table 3. The first category consists of countries with a population over 50 million. This category comprises seven countries, such as the United States, Brazil, and Japan. The second category consists of countries with a moderate population size, ranging from 10 million to 50 million. This category includes seven countries, namely Spain, Canada, and Australia. The third category comprises countries with a population size ranging from 5 million to 10 million, such as Sweden, Austria, and Switzerland. The fourth category comprises countries with a population size of fewer than 5 million, including Costa Rica, Ireland, and Slovenia. Descriptive statistics were performed on the pertinent variables of nations in each category, comparing the median values of each indicator (refer to Tables 4 and 5). In comparison, the third group of countries reached their peak carbon emissions earlier, with 67% of these nations hitting their peak by 2000 or earlier.

**Table 4:** Classification of selected countries that have peaked in carbon emissions by population size.

Category	Country	Year of Carbon Peak	Total Population
Category 1 (Population ≥ 50 million)	United States	2007	301231207
	Brazil	2004	184006479
	Japan	2013	127445000
	France	1991	58559309
	Italy	2007	58438310
	United Kingdom	1991	57424897
	South Korea	2013	50428893
Category 2 (10 million ≤ Population < 50 million)	Spain	2007	45226803
	Canada	2007	32889025
	Australia	2006	20697900
	Netherlands	1996	15530498
	Greece	2007	11048473
	Portugal	2005	10503330
	Belgium	1996	10156637
Category 3 (5 million ≤ Population < 10 million)	Sweden	1993	8718561
	Austria	2003	8121423
	Switzerland	2000	7184250
	Denmark	1996	5263074
	Finland	1994	5088333
Category 4 (Population < 5 million)	Costa Rica	1999	3885428
	Ireland	2001	3866243
	Slovenia	2008	2021316
	Cyprus	2008	1081568
	Iceland	2008	317414

**Table 5:** Median indicators for countries by population size.

Median Indicator	Urbanization Rate (%)	GDP per Capita (USD)	Carbon Emission per Unit GDP (kg/USD)	Per Capita CO_2_ Emissions (tons)	Share of Agriculture in Value Added (%)	Share of Industry in Value Added (%)	Energy Intensity of GDP (USD/kg)	Fossil Fuel Energy Consumption (%)	Arable Land Area (%)	Forest Area (%)
Category 1	80.27	27182.70	0.35	9.89	1.90	24.31	8.38	85.62	17.66	33.53
Category 2	77.74	29006.80	0.44	11.20	2.51	25.64	8.11	84.12	19.92	29.57
Category 3	**80.69**	**32294.00**	**0.31**	**9.17**	**2.79**	**25.54**	**10.55**	**56.89**	**10.34**	**46.57**
Category 4	59.40	28282.40	0.29	7.77	2.09	25.73	12.30	69.51	8.92	18.68

### (1) Levels of economic and urbanization development

**Fig 1:**
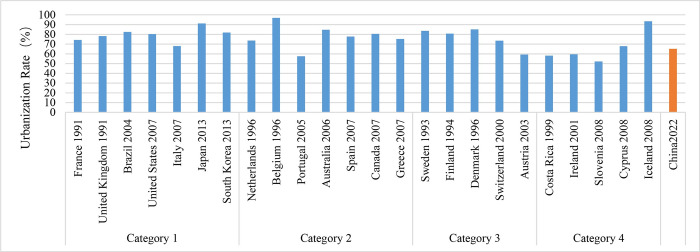
The urbanization rate of countries with carbon peaks.

**Fig 2:**
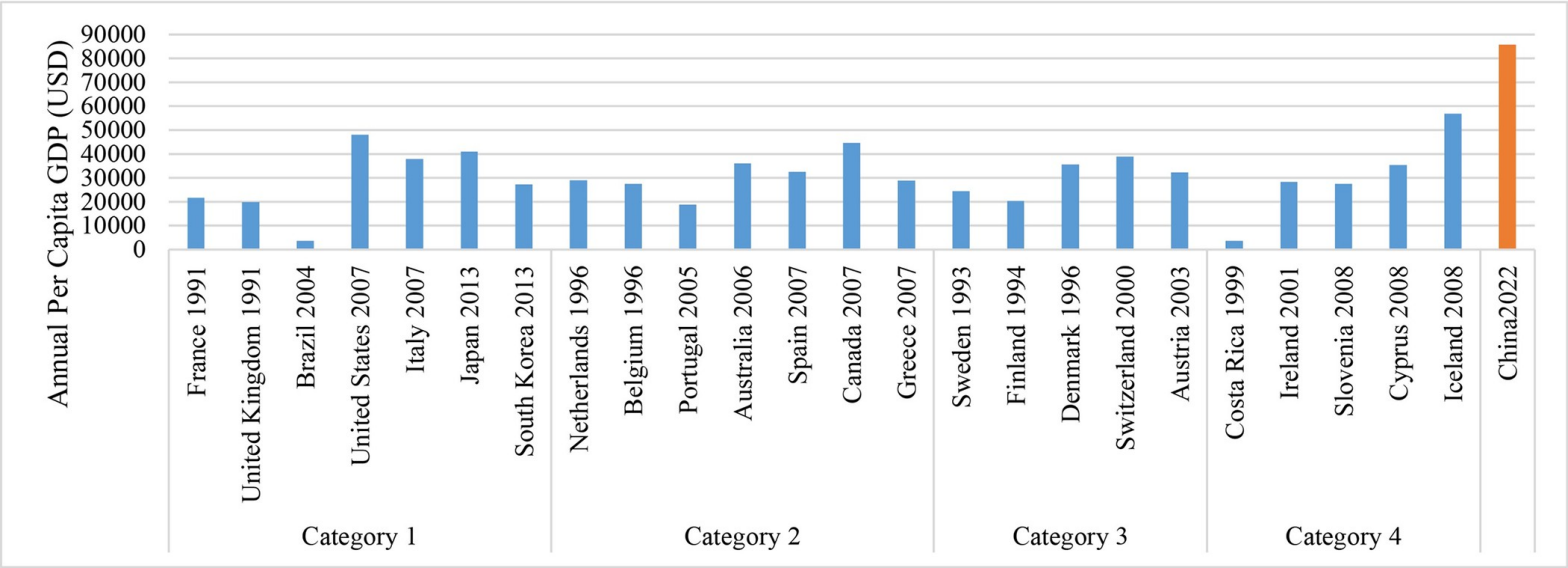
GDP per capita in the year when carbon peaked.

The nations classified as Category 1, which have the greatest population, as well as those in Category 3 with relatively small population, have urbanization rates above 80%, as indicated in Fig 1. The GDP per capita for Category 1 countries is $27,182.7, whereas for Category 3 countries it is $32,294.0. Countries in Category 2, characterized by moderate population levels, exhibit a median urbanization rate of 77.74% and a GDP per capita of $29,006.8. Category 4 countries, which are the smallest in size, exhibit a significantly low urbanization rate of approximately 60% and possess a GDP per capita of $28,282.4. With the exception of Brazil and Costa Rica, all the other countries mentioned are developed nations characterized by advanced levels of economic and social development. During the period when carbon peaking was achieved, urbanization levels were generally high, indicating that urbanization is already at a relatively mature stage. The smallest countries, however, exhibited much lower rates of urbanization compared to the rest. Regarding GDP per capita, countries that reached their highest levels of carbon emissions in the 21st century, except for Brazil, generally had a value above $30,000. The United States, Canada, and Iceland even surpassed $50,000. On the other hand, countries that peaked in carbon emissions in the previous century, with the exception of Costa Rica, maintained a level of around $20,000, as shown in Fig 2.

By comparing the urbanization rates and GDP per capita of various countries when they reached their highest levels of carbon emissions, a pattern emerges: a high urbanization rate and a stable growth rate often indicate a country’s urbanization process in a mature stage. This is often accompanied by lower carbon emissions associated with urban construction, which in turn facilitates the peak in carbon emissions. Furthermore, in nations with higher population sizes, the influence of urbanization rates on expediting the peak of carbon emissions seems to be more substantial. Moreover, a greater Gross Domestic Product (GDP) per capita indicates a better degree of economic advancement, which has a beneficial impact on reaching the peak of carbon emissions. Nevertheless, there is not always a clear and direct relationship between the time point of carbon emissions reaching their peak and the Gross Domestic Product (GDP) per person. Within nations that share comparable levels of economic development, the zenith of carbon emissions may be delayed until the economy exhibits greater strength. This implies that simply achieving a particular GDP per capita threshold is insufficient for reaching the maximum amount of carbon emissions. It is also necessary to take into account the economic conditions and environmental policies in place at that moment.

Furthermore, international trade has had a significant impact on the increase in global carbon emissions, primarily due to the shift in production activities from countries with higher environmental standards to those with lower standards [19]. This shift is attributed to the lax environmental regulations and monitoring systems in the latter, which reduce production costs while increasing emissions of pollutants and greenhouse gases [20]. Additionally, the transportation of goods from these producing countries to distant consumer nations further escalates carbon emissions [21]. This phenomenon, often referred to as "carbon leakage," reveals a crucial reality: despite the implementation of carbon reduction measures by some nations, global total carbon emissions may not have significantly decreased due to the inconsistency in global environmental standards [22]. Moreover, the optimization of global supply chains further encourages firms to produce in countries with lower environmental requirements to reduce costs, not only exacerbating carbon emissions in these countries but also potentially triggering a "race to the bottom" in environmental standards [23]. This trend underscores the importance of international cooperation in harmonizing environmental standards and promoting global sustainable development. Technological innovation also plays a crucial role in reducing carbon emissions and promoting sustainable development. Recent studies continuously highlight the significant contributions of various innovative technologies towards achieving carbon reduction goals, particularly in the fields of renewable energy, energy efficiency, carbon capture and storage (CCS), smart grids, and electric vehicles. Research indicates that renewable energy technologies significantly reduce costs and enhance carbon reduction potential through improved material efficiency [24]. Similarly, energy efficiency technologies optimize energy performance in the building and industrial sectors through innovative thermal insulation materials and smart energy management systems [25, 26]. The development of CCS and smart grid technologies also plays a vital role in industrial processes and improving energy distribution efficiency [27]. Furthermore, the proliferation of electric vehicle technology is crucial for reducing carbon emissions in the transportation sector, necessitating synchronized developments in policy and infrastructure [28]. These technological innovations underscore the potential impacts of cross-disciplinary technological advancements on global carbon reduction, highlighting the critical role of ongoing innovation and policy support in achieving global carbon neutrality goals.

China’s urbanization rate in 2021 stood at 62.51%, suggesting that the country is currently in the process of urban development and requires further infrastructure development to accommodate further urban growth. In the same year, China’s GDP per capita was at $12,556.33, which was considerably lower in comparison to nations that had already reached their peak in carbon emissions. Compared to these countries, China must raise its urbanization rate to a minimum of 70% and boost its GDP per capita by two to three times its current level. China faces a huge challenge in balancing high-quality economic growth with the reduction of carbon emissions as it strives to reach its peak in carbon emissions.

### (2) Levels of carbon emissions

**Fig 3:**
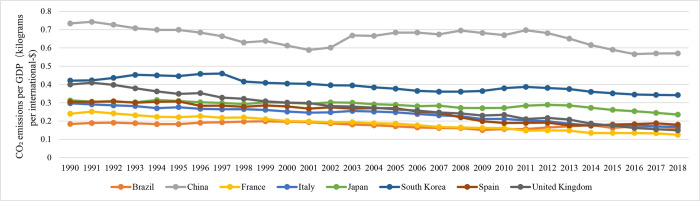
Carbon emissions per unit GDP of typical countries with carbon peaks and China.

**Fig 4:**
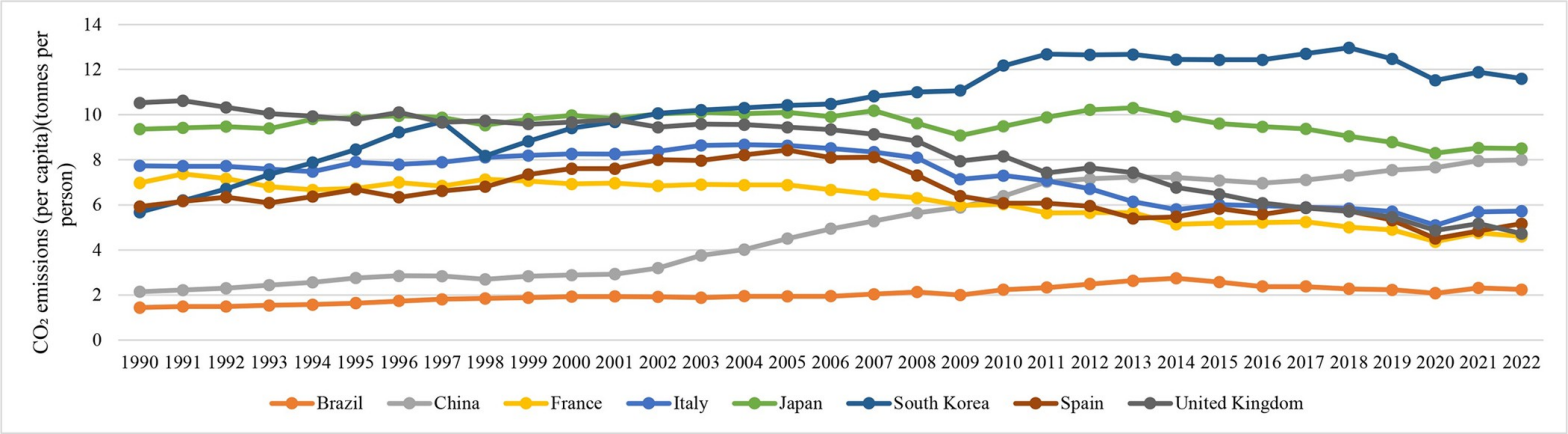
Carbon emissions per capita in typical countries with carbon peaks and China.

Medium-sized population countries have the highest carbon emissions per unit of GDP, measuring 0.439 kg/USD. These countries also have the highest per capita carbon emissions, which amount to 11.199 tons. On the other hand, both the most and least populous countries have carbon emissions per unit of GDP that are greater than 0.3 kg/USD, and their per capita emissions are higher than 9 tons. The countries with the smallest populations show the lowest carbon intensity, with emissions per unit of GDP at 0.291 kg/USD and per capita emissions at 7.767 tons. The carbon intensity of GDP for countries at their peak is approximately 0.3–0.4 kg/USD, whereas per capita emissions range from 9–12 tons (Fig 3). Research conducted in several nations, including Brazil, Spain, France, the United Kingdom, Italy, Japan, and South Korea, has shown a consistent decrease in carbon emissions per unit of GDP after reaching their highest point (Fig 4). Additionally, per capita carbon emissions tend to reach their peak earlier than the overall carbon emissions peak. This indicates the consequences of a decrease in economic growth and the population entering a period of moderate and stable growth.

In 2019, China’s carbon emissions per unit of GDP decreased to 0.457 kg/USD, consistently trending downwards. The per capita carbon emissions stood at 7.606 tons, without any clear signs of reaching a peak. This highlights the necessity of studying and implementing policies concerning economic, social, and industrial energy systems to reduce carbon emissions and expedite the attainment of carbon peaking goals.

### (3) Industrial structure

**Fig 5:**
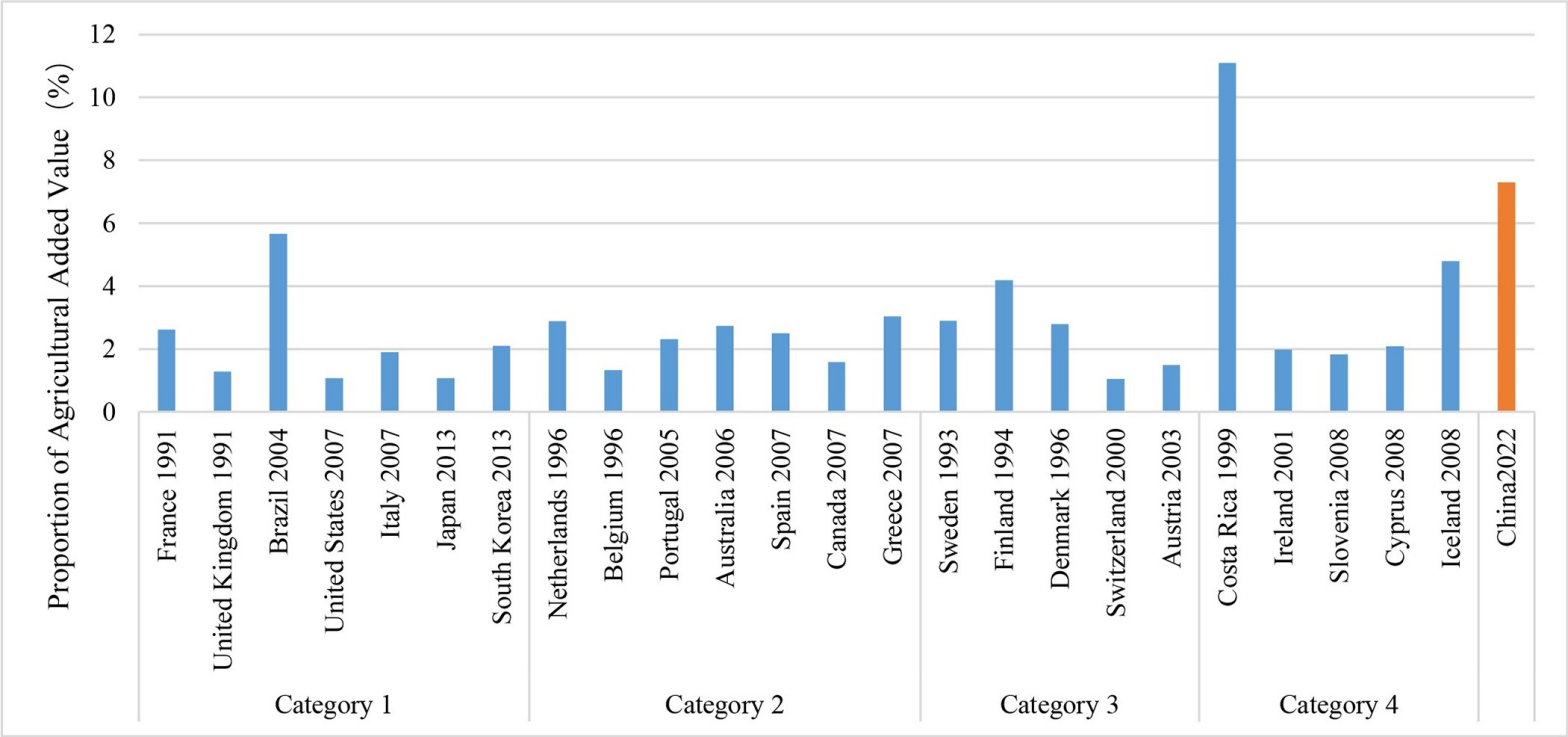
The proportion of agricultural added value in countries that have reached their carbon peaks.

**Fig 6:**
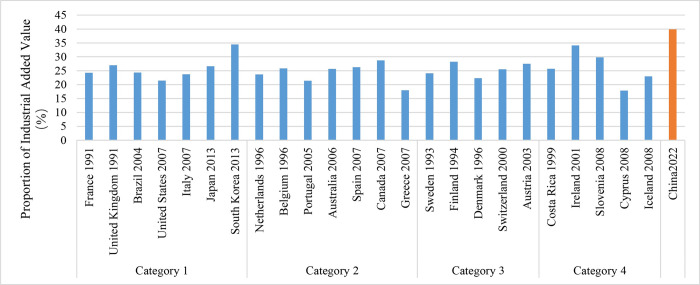
The proportion of industrial added value in countries that have reached their carbon peaks.

During the pursuit of carbon peaking, countries with different population sizes have typically transitioned into the post-industrial phase. This period is defined by a minimal contribution from agriculture, a moderate contribution from industry, and the largest proportion coming from the service sector, which exceeds 50%. In highly populated countries, the agricultural sector contributes just 1.90% to the Gross Domestic Product (GDP), while the industrial sector contributes 24.31%, which is lower compared to other types of countries (Figs 5 and 6). These nations exhibit a consistent decline in the relative importance of agriculture and industry in their industrial structure, accompanied by a corresponding growth in the service sector. When carbon emissions reach their peak, the industrial structure has become relatively stable, completing a shift from heavy industry to contemporary service sectors. After reaching the top, the industrial structure undergoes minor variation.

This prevailing pattern in the evolution of industrial frameworks indicates that a steady sector controlled by service-based activities has enabled the attainment of carbon peaking. This phenomenon is observed in all countries that have reached their peak, and there is no notable connection to the size of the country’s population. Previous studies suggest that countries and regions that have reached their peak can be categorized into two scenarios: "natural peaking" and "economically driven peaking." For example, countries such as the United States and Italy, which have a large share of their economy in the service sector, have achieved fast economic progress and have successfully separated economic growth from carbon emissions. Countries like Poland and Romania had a peak in carbon emissions mostly because of historical factors. And countries like Norway, Finland, and Sweden have successfully decreased their emissions by relying heavily on renewable energy sources [7].

In contrast, agriculture and industry in China accounted for 7.27% and 39.43% of its GDP in 2021, respectively, suggesting that China is currently in the middle of industrial revolution. China is striving to transform the extensive economic mode into an intensive one This shift is based on a development model that focuses on minimizing resource input and consumption while maximizing efficiency and quality. Industrial transformation is still ongoing, and it is necessary to put a greater emphasis on the service sector to match the typical trend seen in nations that have reached carbon peaking. Existing research highlights several critical issues that must be addressed during the transition from agriculture to the secondary and tertiary sectors. For instance, energy poverty is notably more severe in agricultural communities. Studies on multidimensional poverty reveal that education and health are crucial dimensions in alleviating poverty, providing valuable insights for the formulation of effective environmental policies. These observations are essential for guiding strategic developments during industrial transformation to ensure socio-economic benefits align with environmental goals [29, 30].

### (4) Energy consumption

**Fig 7:**
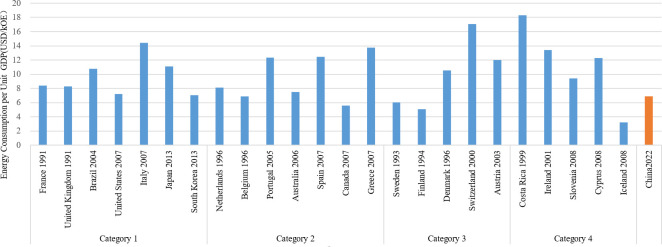
Energy consumption per unit GDP of countries that have reached their carbon peaks.

**Fig 8:**
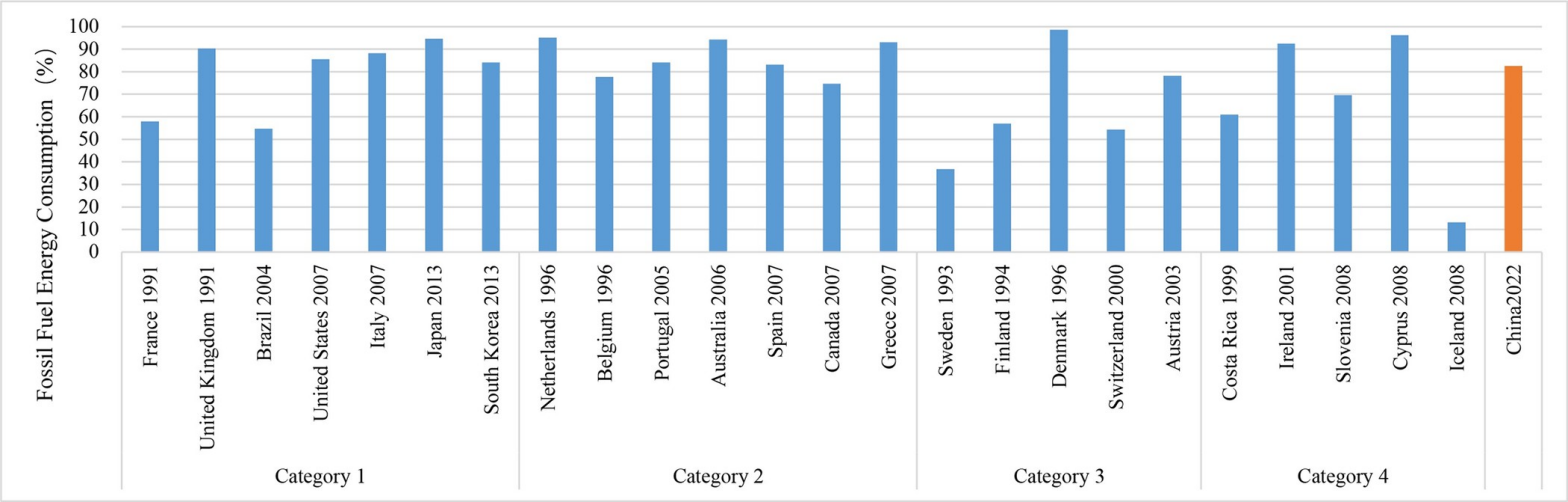
Proportion of fossil fuel consumption in countries that have reached their carbon peaks.

Research reveals that nations with smaller populations tend to have greater energy consumption per unit of GDP, with Category 4 countries demonstrating the highest consumption at $12.30/USD/kg (Fig 7). Countries with larger populations exhibit a slightly lower energy consumption per unit of GDP. Specifically, Categories 1 and 2 have energy consumption rates that exceed $8.00/USD/kg, whereas Category 3 surpasses $10.00/USD/kg. In Categories 1 and 2, the proportion of energy consumption from fossil fuels exceeds 84%, which is greater than in Categories 3 and 4 (Fig 8). Notably, nations such as Sweden and Finland have a fossil fuel energy consumption below 60%. Moreover, in recent years, there has been a general decline in the global share of fossil fuel usage.

Energy intensity, measured as energy consumption per unit of GDP, serves as an indicator of both the reliance on energy and the effectiveness of economic growth. It is influenced by factors such as population size, industrial composition, and technological advancements. This aligns with recent findings which demonstrate that technological advancements play a significant role in enhancing environmental quality, supporting the potential for technology-driven environmental policies [31]. Although a higher energy consumption per unit can force the implementation of carbon reduction measures, there is presently no universally established limit or standard that specifies the maximum energy consumption per unit of GDP. The percentage of fossil fuel consumption provides insight into the composition of energy usage, with an approximate average of 79% across countries that have reached their peak, indicating that fossil fuels continue to hold a significant position in global energy consumption. As a reaction to the energy crisis, countries are making progress in energy transitions by decreasing the reliance on fossil fuels and exploring alternative energy sources.

China’s present energy intensity, measured as energy consumption per unit of GDP, stands at around $6.00/USD/kg. This figure is slightly lower than that of nations that have reached their peak. Additionally, the proportion of fossil fuels consumption exceeds 80%, which is similar to that of countries that have already peaked. Research indicates that increasing renewable energy consumption significantly reduces CO_2_ emissions, offering important policy insights compared to current energy policies in China [32]. Amidst the worldwide shift towards renewable energy and increased focus on energy efficiency, China faces the crucial task of pursuing a sustainable, low-carbon energy strategy and enhancing energy utilization efficiency in order to achieve its carbon neutrality objectives.

### (5) Ecological environment

**Fig 9:**
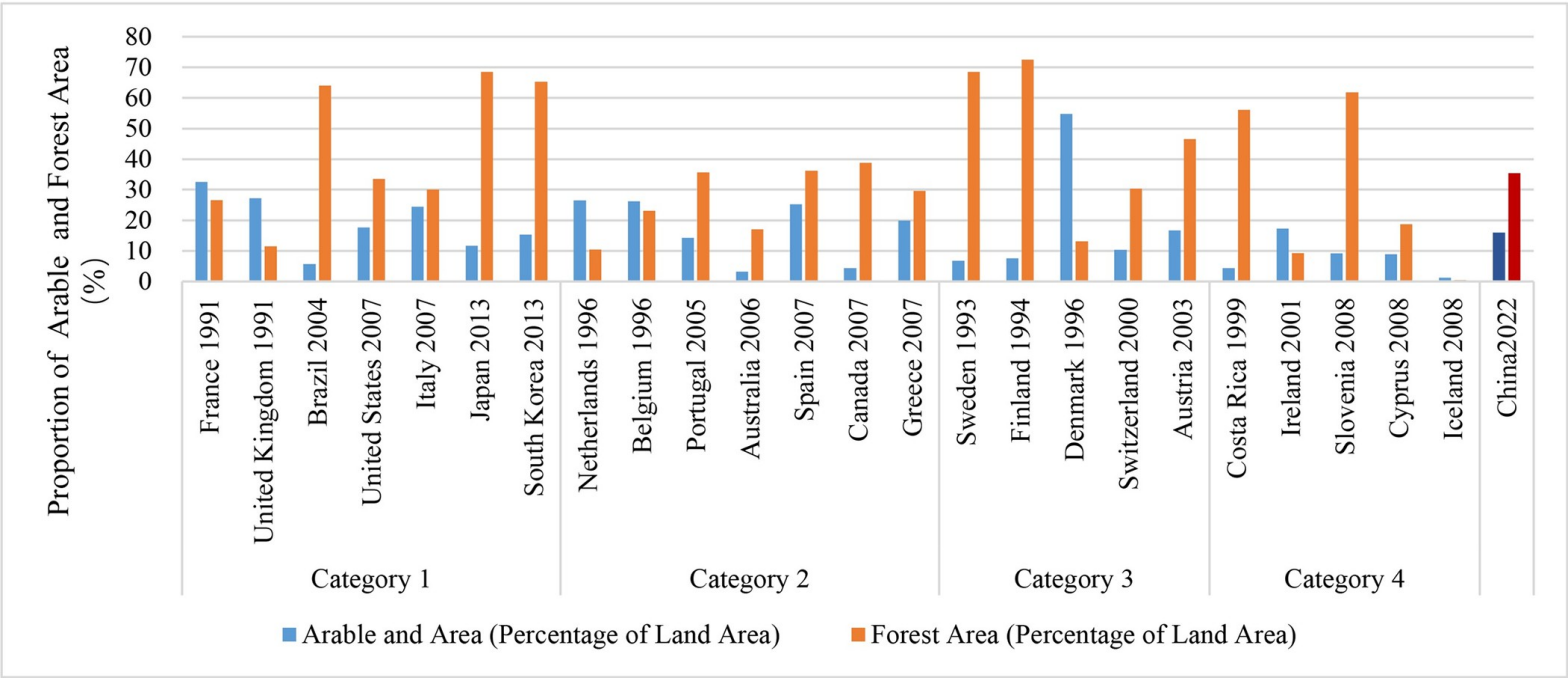
The proportion of arable and forest land in countries that have reached their carbon peaks.

The distribution of arable land and forest regions in a country is influenced by its geographic position and natural resources, which indicate the superiority of its ecological foundation. An optimal ecological environment enhances the capacity for carbon sinks, therefore counterbalancing carbon emissions. The median proportion of arable land among countries that have reached their peak varies from 17% to 20% (Fig 9). Australia has the lowest percentage at just 3.15%, while Denmark has the highest at 54.73%. The median proportion of forested land, expressed as a percentage of the total land area, ranges from 18% to 47%. Iceland has the lowest percentage, with only 0.42% of its land covered by forests, while Finland has the greatest percentage, with 72.57% of its territory covered by forests.

China currently possesses around 12.7% of arable land and 23.4% of forest area. The country prioritizes the safeguarding of fertile land and enforces tight regulations to prevent its conversion to non-fertile purposes. Additionally, it has established a compensation mechanism for cases where land is occupied. Measures have been implemented to limit deforestation, including measures aimed at promoting tree planting, afforestation, and forest conservation to increase forest area. The "Forestry Law of the People’s Republic of China" and other similar policies and regulations are implemented to enhance the consciousness and safeguard the ecological environment.

### 3.4 Overview of fundamental attributes of carbon-peaked nations

From the perspective of economic and urbanization development, countries that have successfully reached carbon peaking tend to have a superior economic status within their respective categories, whether they are classified as developed or developing. There is a strong association between the year in which carbon emissions peak and the GDP per capita in these nations. Specifically, countries that have a later year of carbon peaking tend to have a higher GDP per capita, showing a close correlation with the development features of the age. Following the peak in carbon emissions, the economic growth rate of these countries tend to exhibits a decelerating pattern. Carbon-peaked countries have achieved high levels of urbanization, often surpassing 70%. This suggests a decreased need for heavy industrial goods in their urban development.

From the perspective of carbon emission levels, there is a correlation between carbon emissions and a country’s economic growth rate as well as population expansion. Specifically, per-unit GDP carbon emissions tend to decrease after reaching the peak of carbon emissions. The maximum level of carbon emissions per person typically occurs slightly earlier than the maximum level of overall carbon emissions. These findings indicate that once carbon peaking is reached, the country’s economic growth rate has become stable, and population growth has transitioned into a phase of moderate to slow development.

From the perspective of industrial structure transformation, countries that have reached carbon peaking are typically in the post-industrial period. These countries have a minimal share of agriculture, a modest share of industry, and a dominant service sector that exceeds 50%. The industrial structure changes in these countries demonstrate an annual decline in the proportion of added value from agriculture and industry, accompanied by a progressive rise in the contributed value of the service sector. This signifies a transition from the heavy industry to the high-end manufacturing and the contemporary services, which in turn enables an earlier attainment of the peak in carbon emissions.

From the perspective of energy consumption structure, countries that have already reached their carbon peak have a high degree of dependence on energy as well as a high utilization efficiency of energy. The average energy consumption per unit of GDP ranges between 9 to 11 US dollars per kilogram, with fossil fuel consumption accounting for a major proportion, averaging 79%. Following the peak of carbon emissions, there is a gradual improvement in energy efficiency, a decrease in energy consumption intensity, and an optimization of the energy structure by reducing coal usage and increasing the utilization of low-carbon intensity energy sources such as natural gas. This promotes the development of a sustainable and low-carbon energy system.

From the perspective of ecological environment. In countries that have achieved carbon peaking, the correlation between forest and arable land areas and carbon peaking is not significant Nevertheless, due to policy limitations, an expanding array of nations are prioritizing the safeguarding of cultivable land and forests, acknowledging their role in augmenting natural carbon sinks and thus advocating for the significance of low-carbon urban development.

The aforementioned research demonstrates that several factors, including economic development, urbanization, shifts in industrial composition, improvements in energy consumption patterns, and preservation of the ecological environment, have a substantial influence on a nation’s ability to reach carbon peaking. These factors interact and jointly promote the country’s shift towards low-carbon growth.

## 4. Discussion

### 4.1 Key strategies for mitigating carbon emissions in China

China, in comparison to countries that have already reached their highest levels of carbon emissions, is still relatively weak in economic growth, urbanization, changes in industrial structure, and energy composition. The nation is currently making its efforts to reach its carbon peaking and carbon neutrality objectives. In order to promote the achievement of carbon peak, a number of measures have been implemented, notably the "14th Five-Year Plan" released in 2021. This plan emphasizes the need to expedite the establishment of an environmentally friendly, low-emission, and circular economic model, as well as the advancement of a revolution in energy generation and utilization. In July of the same year, China launched the national carbon emissions rights trading market, which presently encompasses the power industry and has plans to eventually expand to other major businesses that emit carbon. In September, the National Development and Reform Commission introduced an energy consumption dual-control system, which established specific targets for both total energy consumption and intensity, to regulate and limit projects that consume large amounts of energy. At the national level, there is a strong push to support the adoption of new energy vehicles and the advancement of renewable energy. The focus is on promoting the growth and usage of renewable energy sources such as wind and solar power.

### 4.2 Trends in policy evolution in carbon-peaked countries

Examining the duration and approaches of carbon reduction policies proposed and implemented by carbon peak countries provides significant knowledge for the creation of China’s own carbon reduction policies. In order to effectively utilize international carbon peaking strategies and propose policy recommendations for China’s carbon peak, this article classifies the carbon reduction policies of different nations into three primary categories and seven sub-categories (Table 6). Additionally, it highlights the changing patterns of various types of carbon reduction policies over time. The development and implementation of these policies are intricately connected to the advancement of the global climate change agenda, as well as factors such as a country’s development stage (which is reflected in technological progress and economic conditions) and political determination, as previously discussed.

**Table 6:** Classification of carbon reduction policies.

Major Category	Subcategory	Policy Introduction Stage	Description	Exemplary Countries/Regions
Policy, Legal Frameworks, and Institutional Mechanisms	Laws and Regulatory Systems	Later Stage	Establishment of legal frameworks and regulations to provide legal support and enforcement for carbon reduction targets. This includes carbon emissions regulation, mandatory emission reporting, and the development and implementation of environmental standards.	■United Kingdom (peaked in 1991) with the Climate Change Act of 2008, setting specific greenhouse gas reduction targets and timelines. ■France (peaked in 1991) with the Energy Transition Law of 2015, outlining a series of climate and energy goals, including reducing fossil fuel consumption and increasing renewable energy shares.
	Carbon Pricing and Market Mechanisms	Mid to Late Stage	Inclusion of carbon taxes and carbon trading systems. Carbon taxes impose charges on carbon dioxide emissions from businesses and individuals to curb emissions by increasing the cost of carbon emissions. Carbon trading involves setting carbon emission quotas and allowing trading within and beyond these quotas to reduce overall carbon emissions through market mechanisms.	■European Union with the EU Emissions Trading System (EU ETS) initiated in 2005, the world’s first and largest carbon emissions trading market. ■Canada (peaked in 2007) implemented a nationwide carbon pricing strategy in 2019, including carbon taxes and emission trading systems, aimed at encouraging greenhouse gas emission reductions.
	International Cooperation and Multilateral Mechanisms	Mid to Late Stage	Promotion of technology transfer, financial flows, and experience sharing through international agreements and cooperation projects to jointly address global climate change challenges.	■Kyoto Protocol signed in 1997 and effective in 2005, aiming to address global climate change by setting specific greenhouse gas reduction targets. ■Paris Agreement reached in December 2015 during COP21, requiring signatories to submit their Nationally Determined Contributions (NDCs) pledging to reduce greenhouse gas emissions. ■EU Carbon Border Adjustment Mechanism (CBAM) proposed in December 2019 as part of the European Green Deal.
Technology Innovation and Efficiency Improvement	**Energy Structure Adjustment Policies**	Mid to Late Stage	Aimed at adjusting the energy consumption structure, reducing dependence on fossil fuels (e.g., coal, oil, and natural gas), and increasing the share of clean energy sources (e.g., wind, solar, hydro, and nuclear). These policies include but are not limited to increasing the proportion of renewable energy in power generation, supporting nuclear energy development, and limiting or gradually phasing out fossil fuel power generation.	■Germany (peaked in 1991) with energy transition policies initiated in the early 2000s, aiming to increase the share of renewable energy in electricity consumption to over 80% by 2050. ■China, in its 14th Five-Year Plan, proposed accelerating the construction of a new energy system dominated by new energy sources, promoting the scale development of wind and solar power generation.
	**Energy Efficiency Improvement Policies**	Early to Late Stage	Aimed at reducing energy consumption and carbon emissions by enhancing energy use efficiency. This includes policies related to building energy savings, industrial energy savings, transportation energy savings, etc., such as promoting energy-efficient building standards, improving industrial production efficiency, developing public transportation, and new energy vehicles.	■Japan (peaked in 2013) with the "Top Runner" energy efficiency standard established in 1999, setting strict energy efficiency standards for various products and equipment, leading market upgrades. ■The United States (peaked in 2007) with Energy Efficiency Resource Standards (EERS) implemented by different states at different times, requiring utilities to improve a certain percentage of energy efficiency annually.
	**R&D and Technological Innovation Support Policies**	Early to Late Stage	Aimed at supporting the research, development, and promotion of low-carbon and clean energy technologies, including government-funded research projects, technological innovation awards, and subsidies.	■South Korea’s Green Growth Strategy proposed in 2008, with increased investment in green technologies in the following years, encouraging the development and commercialization of low-carbon technologies. ■Norway’s investment in Carbon Capture and Storage (CCS) technology since the 1990s, particularly in the application in oil extraction and industrial sectors.
Natural Resources and Environmental Management	**Forestry and Land Use Policies**	Early to Late Stage	Aimed at increasing carbon sinks by improving forest coverage, implementing sustainable land management and agricultural practices. This includes afforestation, avoiding deforestation, and improving land use methods.	■Brazil (peaked in 2004) with the Amazon Fund established in 2008, supporting the protection and sustainable use of the Amazon rainforest to reduce deforestation and carbon emissions. ■Indonesia’s implementation of sustainable palm oil production policies since 2006, focusing on good agricultural practices, protecting high-carbon storage forests and wetlands, and increasing transparency and accountability in the industry chain to reduce environmental damage and greenhouse gas emissions.

Examining the date of the adoption and execution of carbon reduction programs globally demonstrates that policies of different types have had distinct focuses and rates of progress throughout different historical epochs. The development of these policies is influenced by the worldwide advancements in tackling climate change, the extent of economic and social growth, the capacity for technical innovation, and shifts in the international political and economic environment. The process of evolution can be categorized into five distinct stages:

Initial Phase (Late 1980s to 1990s): At first, the main objective was to enhance energy efficiency and establish fundamental environmental safeguards such as increasing energy efficiency requirements in the construction and industrial sectors, as well as encouraging energy-saving technology and goods. The implementation of strategies to enhance natural carbon sinks, such as the preservation of forests and the establishment of new forests, also started to receive recognition.

Post-1997 marks the second stage which is the era of the Kyoto Protocol. Upon the ratification of the Kyoto Protocol, the international community acknowledged and endorsed carbon trading and market systems, including the Clean Development Mechanism (CDM). Over time, laws and regulatory mechanisms were implemented, leading several countries to create specific national targets and restrictions on carbon emissions.

The third stage is from the early to mid-2000s, during which there was an increase in support for renewable energy through the implementation of various measures such as subsidies, tax incentives, and renewable energy generation quota schemes. Energy structure adjustment policies gradually became the focus, especially in some developed countries, where reducing dependence on fossil fuels began to be emphasized.

The fourth stage is the "Paris Agreement period" (after 2015). With the adoption of the Paris Agreement, governments have prioritized the fulfillment of emission reduction obligations, hence intensifying global endeavors to decrease emissions. Carbon pricing and market mechanisms have been expanded, with a growing number of governments and regions implementing or intending to implement carbon pricing mechanisms, such as carbon taxes and regional carbon trading markets. Policies supporting research and development (R&D) and technological innovation have gained significance, particularly in terms of investing in advanced technologies such as green hydrogen, Carbon Capture and Storage (CCS), and Carbon Capture and Utilization (CCU).

The fifth stage is the new era since the early 2020s. In this stage, the increasing urgency of climate change has led to a stronger support for policies that aim to speed up the transition to clean energy, make adjustments to industrial structure, and promote the use of environmentally friendly and low-carbon technologies. International cooperation and multilateral frameworks play a crucial role in promoting global emission reductions. This includes providing support to developing nations in their attempts to reduce emissions through technology transfer and green finance.

### 4.3 Discussion on policy recommendations for carbon reduction in China

Overall, in the context of economic development and urbanization, China must prioritize the advancement of the urbanization process, elevate urbanization levels, and expedite economic growth and industrial upgrading in order to raise GDP per capita. In terms of industrial structure, the development of modern service industries should be strengthened to facilitate the transformation of industries towards servitization. Efforts must be made in the energy sector to accelerate the investigation of low-carbon and sustainable energy transition solutions, enhance energy efficiency, and optimize the economic advantages of energy use. Regarding ecological and environmental protection, it is crucial to prioritize the enhancement and improvement of ecosystem management. This includes extending ecological spaces and boosting their quality in order to boost urban carbon sequestration capacity.

In order to reach the point of carbon peaking, China has implemented various measures including the establishment of a national carbon emissions trading market, the implementation of a dual-control system for energy consumption, and the introduction of policies that promote adjustments in energy structure. Nevertheless, in comparison to nations that have already reached their highest point, there remains a deficiency in the development and execution of precise measures pertaining to low-carbon technologies, carbon levies, global collaboration, regulatory frameworks, and the promotion of carbon sinks. How to reasonably arrange the timing of policy introduction and implementation to minimize any negative impact on the pace of economic development while simultaneously advancing carbon peaking, is a subject that merits thorough examination. It is crucial to acknowledge that most developed countries that have reached the peak of carbon emissions mostly experienced a shift in their development trajectory. Initially, their rapid development of productivity were driven by the large-scale application of high-carbon energy sources during the Second Industrial Revolution. Later, they transitioned to the Third Industrial Revolution, where emerging science and technology played a central role in driving their development [11]_._ Carbon emissions only began to decline after experiencing a period of growth plateau, and economic development gradually decoupled from carbon emissions. Implementing carbon limitations too early may impede the progress of a country’s economy, and the imposition of carbon taxes is linked to the level of development; collecting taxes prematurely might impact the motivation of businesses. In addition, Low-carbon technology innovation is widely acknowledged for its crucial role in decreasing emissions. The implementation of low-carbon technology is regarded the primary approach to reach carbon dioxide emission reduction goals [4].

Thus, it is advisable to implement low-carbon policies in a phased manner. Initially, the focus should be on increasing public and corporate environmental awareness. In the medium term, the emphasis should shift towards promoting low-carbon technologies and making adjustments to the energy structure. Finally, in the later stage, it is crucial to strengthen the enforcement of laws and regulations and enhance international cooperation. In terms of the top-level design, it is crucial to establish a comprehensive and well-defined roadmap and timeline that specifies the short-term, medium-term, and long-term objectives and identifies the essential policies and measures that will be implemented at each step. Policy formation should be synchronized with environmental policies to guarantee that economic policies are congruent with environmental protection policies, promoting and directing low-carbon economic development through fiscal, tax, and financial measures to prevent policy conflicts. It is also crucial to promote research and development (R&D) and the implementation of low-carbon technologies, facilitate the growth of clean energy sectors, enhance energy efficiency, and foster collaboration with technologically advanced countries. Implementing a variety of market mechanisms, such as carbon taxes and green credits, in addition to the carbon emissions trading market, can effectively direct money towards low-carbon projects and technology. Implementing a robust legal, regulatory, and standard framework to tightly control carbon emissions guarantees the successful enforcement of policies. Encouraging the development of policies that support the protection and restoration of natural carbon sinks, such as forests and wetlands, and promoting the establishment of new carbon sink projects, such as urban greening and agricultural carbon sequestration, in order to incentivize carbon sink development [33]^.^ Promoting public engagement involves teaching and increasing public awareness of environmental conservation, fostering participation in low-carbon initiatives across all societal sectors [34, 35]_,_ and cultivating a favorable environment for advancing carbon peaking. It is essential to enhance monitoring and evaluation by implementing a system to monitor and assess carbon emissions and energy consumption. This should be accompanied by regular publication of reports and making necessary adjustments and improvements to associated policies based on the actual outcomes.

### 4.4 Examples of detailed policy recommendations

To further specify our policy recommendations, we can draw upon specific technologies and strategies that have been successfully implemented in diverse geographical contexts. For instance, the German Energiewende-Germany’s comprehensive energy transition initiative—provides a robust model for China. This initiative includes substantial governmental support for the advancement of renewable energy technologies such as wind and solar power. Crucially, the German approach incorporates feedback mechanisms that ensure a seamless integration of technological innovations within market frameworks. Analogously, China could implement incentive structures including tax incentives, initial capital investments, and sustained technical support to foster the commercial scalability of these renewable technologies.

Regarding urban planning strategies, the Singaporean ’Garden City’ vision offers a paradigmatic example of how urban greenery integration can significantly enhance carbon sequestration capacities while simultaneously improving urban microclimates and living standards. Large Chinese metropolises like Beijing and Shanghai might benefit from embedding similar green infrastructure projects within their urban development plans, thereby bolstering sustainability and enhancing residents’ quality of life.

Moreover, the policy frameworks established by Norway to promote electric vehicles (EVs) are particularly instructive. By exempting EVs from certain taxes, offering free parking, and granting access to bus lanes, Norway has dramatically accelerated the adoption of electric vehicles. These initiatives have markedly decreased carbon emissions in the transport sector. By implementing parallel fiscal incentives and municipal supports, China could expedite the adoption of EVs and the electrification of its public transport systems, thus advancing its low-carbon transportation agenda.

### 4.5 Challenges and strategies for low-carbon policy implementation in China

The implementation of low-carbon policy in China encounters complex hurdles, especially in the realms of politics and economics. Politically, the main difficulties stem from the requirement for consistency and the clash of priorities in policy execution. Environmental policy must be harmonized with economic development objectives, requiring a delicate equilibrium between economic expansion and environmental preservation. Furthermore, the different degrees of dedication and allocation of resources by local governments, along with inconsistencies in policy enforcement between central and local authorities, greatly impede the effectiveness of environmental rules. From an economic standpoint, one of the obstacles is the substantial upfront expenditures associated with implementing new energy technologies and energy efficiency initiatives. These technologies face competition from more affordable conventional energy sources, like as coal and natural gas, especially in the early phases. China, being a major global consumer of energy and heavily dependent on coal, faces significant challenges in shifting to a new energy framework. In addition, the transition to new energy sources and the promotion of energy-efficient technology may result in job reductions in conventional sectors like coal mining and heavy industries, affecting workers and their communities. To tackle these difficulties, it is necessary to develop comprehensive strategies that take into account technological, policy, economic, and social factors. This approach is crucial to ensuring that policies are effective and get widespread social support. The government should adopt workforce transition policies and retraining initiatives to minimize the societal costs linked to the energy transition. This comprehensive approach highlights the importance of incorporating various aspects to successfully implement environmentally friendly policies with broad support in China.

Future research should prioritize addressing the political and economic challenges that China faces in the implementation of low-carbon policies to foster sustainable development at the national level. This necessitates a focus on policy integration, aimed at establishing coherence between environmental policies and economic development goals. Additionally, it is essential to tackle the difficulties in coordinating policy execution across central and local governments. Moreover, the importance of incentivizing local governments should be underscored to enhance their motivation and efficacy in enforcing environmental regulations. Economically, research should investigate market-based mechanisms to alleviate the considerable initial costs associated with emerging energy technologies and energy efficiency measures. This includes exploring strategies to reduce dependence on traditional energy sources. Furthermore, considering the impact of technological transitions on employment, it is critical for research to develop effective strategies for workforce adaptation, ensuring a smooth shift from traditional to emerging energy sectors. Further research should also examine the influence of spatial organization on reducing carbon emissions, specifically how urban planning and land use optimization can enhance energy efficiency and promote sustainable transportation and building designs. By concentrating on these comprehensive areas of study, we can effectively tackle the complexities and challenges of implementing low-carbon policies in China, thereby providing scientific support and recommendations for the advancement of China’s environmental policies.

## Supporting information

S1 Data (XLSX)
